# Supplementary material for: Are carbon emissions trading and green financial instruments synergistic? -Comprehensive quantitative research based on content analysis
Source: PLoS One. 2024 Mar 7;19(3):e0298601. doi: 10.1371/journal.pone.0298601 (PMC10919601; doi:10.1371/journal.pone.0298601)
Supplement: S1 Data — (ZIP) [file pone.0298601.s001.zip › Data/地级市Z_F工作报告.zip!/╣π╬≈/└┤▒÷╩╨/└┤╫╘╣1⁄2╓┌║┼í╛┬φ┐╦╩2╛▌═°í┐_2015.docx]

各位代表：

现在，我代表市人民政府向大会作政府工作报告，请予审议，并请市政协委员和列席人员提出意见。

一、2014年工作回顾

过去一年，受宏观经济市场环境影响，我市经济下行压力持续加大，制糖、电力、冶炼等支柱产业生产经营没有根本好转。主导工业产品市场萎缩，价格下跌，企业效益下滑，利税缩减，严重影响财政收入；在建重大项目进入尾声，新增项目少，发展后劲不足。面对严峻复杂的经济形势，在自治区党委、自治区人民政府和市委的坚强领导下，市政府积极谋划，全力应对，及时采取系列强力措施，按照稳中求进的总基调，着力转方式、调结构、扩内需、惠民生、促和谐，保持经济社会平稳健康发展。初步统计，全市地区生产总值完成551.24亿元，增长6.1%；固定资产投资431.18亿元，增长5.1%；财政收入58.11亿元，增长3.5%；社会消费品零售总额134.17亿元，增长11%；城镇居民人均可支配收入25401元，增长7.8%；农民人均纯收入7751元，增长9.4%。

（一）各项改革稳步推进。一是行政审批制度改革在全区率先启动。出台了改革总体方案和《工商登记制度改革实施方案》《加强行政审批监管实施意见》等7个配套制度，市本级行政审批事项从368项缩减到148项，减少60%。民间资本投资有了较大提升，市场活力得到有效释放，新增私营企业1727户，增长22.6%。二是国资和国有投融资企业改革稳步推进。将10家市属企业合并重组为三大集团公司，同时整合组建了市水务集团公司，实现了资源优化配置。实质性启动广铁公司改革。三是政府职能转变和机构改革有序推进。全面完成市城市管理局、卫生和计生、文化新闻出版广电、食品药品监督等部门职能和机构整合，组织结构进一步优化。四是金融财政体制改革有效推进。采取停止新BT项目、集中融资财力、统一土地出让标准等九项措施，缓解债务危机，共化解政府性债务87亿元。农村金融服务覆盖到乡镇，并逐步延伸到村屯。五是议事规则和重大制度进一步完善。成立了市城乡规划审批委员会、土地开发利用委员会、财政金融工作委员会，强化市编委会的议事职能。出台了《加快新型工业化实现跨越发展的决定》《关于加快改革创新全面振兴教育的决定》《来宾市城区供地规模控制及价格管理办法（试行）》等制度。六是农村土地制度改革扎实推进。农村土地承包经营权确权登记颁证试点工作走在全区前列，自治区在象州县召开工作现场会。同时，城市管理体制机制、公共资源交易体制、新农合支付方式等改革也取得较好成效。

（二）工业经济逆势发展。深入实施工业发展“四大会战”，规上工业总产值完成517.7亿元，增长3.0 %，规上工业增加值增长6.6%。一是“铝电结合”取得重大突破。自治区批复同意在我市开展银海铝直购电试点工作，银海铝业从7月份开始恢复生产，并带动下游铝加工企业11月份全面复产达产。二是工业项目建设加快推进。全市重点推进工业产业项目90个，其中新竣工项目35个，新开工项目45个。合山发电公司“上大压小”第二台机组项目获得自治区发改委核准，广铁公司合金新材料、敏诚矿业公司二期项目通过自治区“两高”审查。三是转方式调结构取得新成效。机械制造、碳酸钙、高新技术等新兴产业发展势头强劲，新兴产业占比达到10%。新签约汽车（机械）零配件制造产业项目15个，涉及投资15亿元；实现碳酸钙产业产值15亿元，中国碳酸钙行业专家组工作年会在我市召开；新增自治区级企业研发中心和技术中心各2家。糖业、铝业、火电、茧丝绸、畜禽养殖、农产品加工等产业链进一步延伸。河南工业园用汽企业达20家，“热电联产”规模居全区之首。淘汰落后产能水泥96.60万吨、铁合金21万吨，关闭整合煤矿井10对。四是园区平台进一步完善。投入7.2亿元推进园区基础设施建设。产城一体化建设进一步加快，来华投资区获国务院侨办授予“全国华人华侨创新创业示范基地”，象州县工业集中区、武宣县工业园区被列为自治区产城互动试点园区。

（三）现代农业加快发展。初步统计全市农林牧渔业总产值219.07亿元，增长2.3%。一是农业产业结构进一步优化。创建粮食高产示范片16个，粮食生产实现“四连增”；建设甘蔗“双高”示范基地19片共2.04万亩，超额完成自治区下达的任务；桑园面积、蚕茧产量实现“三连增”；水果面积增长5.6%，蔬菜增产8.3%；新建千头以上规模养牛场4个，万头养猪场3个。二是农业生产经营方式加快转变。新培育市级龙头企业8家、专业合作社132家、家庭农场111家。建成高标准农田3.56万亩，新增流转土地10.99万亩。农业机械总动力182.30万千瓦，农业生产机械化水平达46.1%。凤凰华侨农场现代农业（核心）示范区通过自治区考评验收，各县（市、区）创建市级现代特色农业（核心）示范区扎实有效。三是农业品牌创建成效明显。兴宾区糖蔗种植基地和凤凰华侨农场获认定为自治区级农业科技园区；全市获得国家、自治区级“三品一标”认定（证）39个；武宣县葵花景观入选“中国美丽田园”。四是农业科技支撑和农产品安全监管能力不断增强。完成全市66个基层农技推广站、农产品质量安全监测站改革和建设，实现自治区、市、县、乡四级监测数据联网。

（四）基础设施不断夯实。桂中治旱乐滩灌区一期工程加快推进，广西首台全断面掘进机（TBM）正式投入使用，完成年度工程投资5.04亿元，累计完成投资13.44亿元，占概算总投资50.8%。二期工程前期工作顺利推进，《可行性研究报告》《水土保持方案报告》等申报材料通过水利部水规总院审查，进入国家水利部审查程序。来宾至武宣高速公路建成通车，来宾至马山、柳州至武宣、梧州至柳州高速公路来宾段建设稳步推进。来宾高铁开通运营，湘桂铁路来宾段电气化改造有效推进。黄金水道建设实现新突破，宾港作业区二期工程建成运营，红水河内河航线断航38年后正式复航，象州港区猛山和钓鱼公作业区、忻城北巷作业区、兴宾港区莆田一期等工程加快推进。

（五）现代服务业持续发展。全年实现第三产业增加值189.59亿元，增长9.3%。商贸物流发展平台日趋完善。南宁百货、南城百货等一批大型商贸企业进驻我市，桂中建材五金家居商贸物流城等重点专业市场相继开业，来宾义乌小商品批发市场建设顺利推进；新增限额以上企业27家；武宣县二塘樟村物流园区、煤炭交易中心等物流项目加快推进。全市公路货运周转量17.44亿吨公里，增长11.4%；水路货运周转量36.71亿吨公里，增长16.5%。信息、拍卖、二手车交易、家政等中介服务加快发展。旅游业发展迅速。金秀县获全区特色旅游名县创建工作进步县，忻城县进入13个备选创建县序列。休闲旅游初具规模，旅游设施不断完善，服务水平进一步提高。全年全市接待游客1218万人次，增长20.5%，旅游总收入75.90亿元，增长37%。金融业平稳发展。2014年末全市银行人民币贷款余额达330.15亿元，增长10.1%。

（六）开放合作不断扩大。积极融入西江经济带和“双核驱动”发展战略，成功将我市102个项目列入自治区西江经济带基础设施建设大会战和“双核驱动”战略重大项目。率先在沿江7市中编制珠江—西江经济带来宾发展规划。承接柳州产业辐射，汽配产业发展势头良好。加深与泰国、印尼等东盟国家的交流合作，第11届中国—东盟博览会期间，签约项目总投资28亿元。招商引资工作扎实有效，实际到位资金315.22亿元。

（七）城镇建设加快推进。获国家列为新型城镇化综合试点城市。一是规划体系不断完善。完成《来宾市中心城区教育用地专项规划》、6个片区控规和4个乡镇、160个村屯的规划编制。二是中心城区辐射带动能力持续增强。桂中水城、教育园区等重大项目稳步推进，城区路网、教文卫体等基础设施和公共服务设施逐步完善。三是人居环境进一步优化。完成迎宾路、桂中大道、维林大道、盘古大道“两纵两横”道路及门边街边、桥下桥边、岸线水边 “三边”生态景观提升改造7.74万平方米，补植城区公共绿地8700平方米，完成政和幼儿园前门规划道路、北二路、梅岭路、城东二路等小街小巷路面硬化1.2万平方米。升级改造老城区西南二路、西南三路及柳来路（来合铁路至铁北路段），有效缓解旧城区出行环境差等老大难问题。合山市、忻城县获“广西园林城市”称号。四是“城市经营管理年”活动取得新成效。老汽车站、老火车站、来宾电厂生活区、豆制品厂、原兴宾区政府等5个片区旧城改造有序推进。完成“数字来宾”二期和“数字化城管系统”项目建设。依法整治新华路、磨东村、政和幼儿园周边、河西二桥头、柳来路等地段违法乱建行为，拆除违法建筑469间5.8万平方米。大力整治乱摆乱卖现象，城区市场秩序进一步好转。五是县城和小城镇建设稳步推进。兴宾城南新区、象州象东新城、武宣城东新区、忻城城南新区、合山“矿山公园”、金秀“公园化”改造等建设加快推进；桐木镇“全区城镇化建设百镇示范工程”深入推进，小平阳镇、凤凰镇、红渡镇、思练镇、石龙镇、武宣镇入选全国重点镇，大湾乡、寺山乡、桥巩乡、城厢乡、河里乡、北泗乡、马坪乡、禄新乡等8个乡获批撤乡设镇。预计全市城镇化率达39%。

（八）绿色发展成效明显。一是“森林城市”创建工作有效推进。深入实施“绿满八桂”工程，植树造林27.93万亩，全民义务植树472万株，森林覆盖率达51.26%，获自治区授予“广西森林城市”称号。二是“美丽来宾·清洁乡村”工作顺利完成。“三清一洁”及铁路公路沿线环境卫生整治成效明显，生产生活环境得到较大改善。启动“美丽来宾·生态乡村”活动。三是节能减排工作深入推进。全市万元GDP能耗和主要污染物排放指标均控制在自治区下达任务指标内。环境空气质量优良天数达351天，地表水和饮用水水源地达标率均为100%。

（九）社会事业全面发展。教育事业加快发展。启动实施教育“十大工程”，学前三年毛入园率达72%，比自治区任务数高12个百分点；义务教育巩固率达84%，超额完成自治区下达的任务；合山市义务教育均衡发展向现代化、信息化提升，忻城县义务教育均衡发展通过自治区评估验收；示范性普通高中实现县域全覆盖；柳州师专高票通过高校设置委员会评审，待教育部批准后将升格为广西科技师范学院，桂林航院来宾校区如期建成办学。科技创新取得新成效。创新型企业及科技企业孵化器建设实现零突破，建成自治区级创新型企业1家，科技企业孵化器2个，全市发明专利拥有量67件，增长137%。文化体育事业蓬勃发展。国家公共文化服务体系示范区建设进一步提升，获列为国家基层综合性文化服务中心建设试点地区；荣获 “全国全民健身示范城市”称号，在武宣县成功举办“全国全民健身示范城市”农民篮球邀请赛；广播电视事业建设和农村公益性电影放映工程扎实推进，完成10座乡镇无线发射台站建设并投入使用，安装开通直播卫星接收设施8155户（套），放映农村公益性电影8765场。卫生计生事业健康发展。自治区卫生城市创建工作稳步推进，市中医院晋升为三级甲等中医医院，市妇幼保健院晋升为三级乙等妇幼保健院，兴宾区人民医院建设顺利推进；新农合参合率达98.68%；计生工作成效明显，人口自然增长率6.53‰，荣获自治区人口计生目标管理工作创新奖，获列为全国幸福家庭创建活动试点市。精准扶贫工作顺利实施，5.27万贫困人口脱贫。社会保障工作扎实有效。城镇新增就业18518人，完成任务的108.9%，城镇登记失业率3.46%，比控制数低0.64个百分点；社会保险、社会救助工作得到加强，发放民政各项社会救助资金3.6亿元，救助困难群众190万人次，率先在全区建立了低收入居民家庭经济状况核对信息平台；全市保障性安居工程开工11393套（户），完成年度任务118.1%，基本建成5290套，完成年度任务150.6%，新增分配入住2723套，完成年度任务108.9%；农村危房改造竣工12000户，竣工率100%。全面完成自治区及市本级为民办实事工程。稳步推进公共法律服务体系建设，获列为全区公共法律服务体系建设试点市。狠抓安全生产，统筹抓好社会稳定，强化社会应急处置，严厉打击各类违法犯罪行为，有效防止重特大事故和群体性事件发生，公众安全感排名保持全区前列，社会和谐稳定。

（十）自身建设不断加强。坚决服从市委领导，坚持重大事项报告制度，自觉接受市人大及其常委会的法律监督、工作监督和市政协的民主监督，发挥人民群众的监督作用，不断提高班子科学决策、依法行政的能力。坚持贯彻落实中央八项规定精神，深入开展党的群众路线教育实践活动，带头纠正“四风”，集中开展公款吃喝、公车私用等专项整治，出台了规范公务接待等一批反“四风”制度，市本级“三公”经费同比下降三分之一，政风社风明显好转。严格执行廉洁从政各项规定，旗帜鲜明地惩治腐败，突出查处了一批大案要案，全市纪检监察机关立案查处各类违纪违法案件272件，挽回直接经济损失3000多万元。坚持以深化改革推进惩防体系建设，进一步规范权力清单、问题清单、责任清单，强化对重点领域和关键岗位的监督，抓好廉政教育和廉政文化建设，营造了风清气正的发展环境。

一年来，我们还加强了与各民主党派、无党派人士、工商联和工会、共青团、妇联、文联的联系，统计、人事、编制、工商、税务、气象、供销、档案、修志、民族宗教、老龄、技术监督、国家安全、决策咨询、新闻出版、外事侨务、防汛抗旱、防震减灾、人民防空、水库移民、社会科学等工作也取得了新成效。国防教育和国防后备力量建设不断加强，全国双拥模范城市创建工作扎实推进。

各位代表，这些成绩是在经济下行的巨大压力下，经全市上下奋力攻坚所取得的，确实来之不易。在此，我代表市人民政府，向全市各族人民，向市人大代表、市政协委员，向驻来宾的中直、区直部门和人民解放军、武警官兵，向各民主党派、无党派人士、工商联、人民团体，向所有关心和支持我市发展的各界人士，表示崇高的敬意和衷心的感谢！

在看到成绩的同时，我们也清醒认识到发展中遇到的困难和问题：实体经济陷入困境，产业竞争力弱、结构不合理，政府债务负担重等问题较为突出，部分主要经济指标没有完成预期目标；一些阻碍经济社会发展的难题仍然存在，用地难、征地难、融资难、财政难等问题还没有得到有效解决；社会不稳定因素仍然存在；保障和改善民生的任务还很艰巨。对这些问题，我们将高度重视，认真研究，采取有力措施加以解决。

二、2015年工作安排

2015年，是全面深化改革的关键之年，是全面推进依法治市的开局之年，也是全面完成“十二五”规划的收官之年。我们将紧紧围绕市三届五次党代会提出的目标要求，抢抓机遇、奋发有为，努力开创经济社会发展新局面。

2015年政府工作的总体要求是：全面贯彻落实党的十八大、十八届三中、四中全会和习近平总书记系列重要讲话精神及中央经济工作会、全区经济工作会精神，以邓小平理论、“三个代表”重要思想、科学发展观为指导，坚持稳中求进工作总基调，坚持以提高经济发展质量和效益为中心，坚持“大兴产业、完善提升、聚集人气、控制规模”和“调整型、追赶型、特色型、绿色型”的发展思路，主动适应经济发展新常态，把转方式调结构放到更加重要的位置，全面深化改革，突出创新驱动，强化风险防控，切实保障和改善民生，统筹推进经济、政治、文化、社会、生态文明建设，全力打造广西“双核驱动”战略重要节点城市。

全年经济社会发展的主要预期目标是：地区生产总值增长6%以上，财政收入增长3%左右，规模以上工业增加值增长8%左右，固定资产投资增长9%左右，社会消费品零售总额增长10%左右，城镇居民人均可支配收入增长8%左右，农民人均纯收入增长9%左右，完成自治区下达的节能减排任务。

提出上述目标，主要是从适应新常态、实现“十二五”目标、全面建成小康社会及经济发展各项支撑条件等因素综合考虑。总体来看，我市仍处于实现跨越发展的战略机遇期。国家重点实施“一带一路”战略、支持中西部地区基础设施建设，宏观经济政策进一步优化，改革开放的红利进一步释放，继续实施积极的财政政策和稳健的货币政策。自治区正在打造“一带一路”有机衔接重要门户，构建西南中南地区开放发展新的战略支点，全面实施“双核驱动”战略，一系列稳增长措施的政策效应将进一步显现。珠江—西江经济带建设上升为国家战略，我市处于西江经济带的重要节点城市，有11个项目列入西江经济带基础设施建设大会战，91个项目列入自治区“双核驱动”战略重大项目建设计划，总投资规模达980亿元；大藤峡水利枢纽工程开工建设，船闸规模将由3000吨级提高到5000吨级，大幅提高我市航道的通航等级，工程还涉及我市移民搬迁安置投资81.2亿元；我市获国家列为新型城镇化综合试点城市，是全区入选的2个市之一，国家将在金融、农民工职业技能培训、城镇保障性住房建设、市政基础设施改造、产业发展和承接产业转移等方面给予倾斜支持。随着交通、水利、工业、旅游、城建、移民等一批重大项目的实施，我市的区位、资源等优势将得到进一步发挥，发展潜力和活力将得到进一步激发。

但是，我们也要看到，目前我市与全国全区一样，经济发展都进入了新常态，呈现出经济增长速度换挡期、结构调整阵痛期、前期刺激政策消化期“三期叠加”的阶段性特征。经济基础薄弱、产业结构单一、抗击市场风险能力弱等固有矛盾仍然非常突出，经济下行压力依然很大。从一产来看，由于甘蔗价格下跌和砍运成本上升，农民种蔗积极性受到影响，甘蔗种植面积和原料蔗产量将有所下降。从二产来看，制糖、电力、冶炼三大支柱产业仍未走出低谷，新增产业项目、培育企业上规潜力有限。从三产来看，受成品油价格下跌、餐饮住宿消费下降、网上购物增加等因素影响，社会消费品零售总额增长将受到制约。从财政收入来看，受来宾B电厂回收、蔗糖增值税减收、营改增等因素影响，原有税源将会减少。实施新《预算法》及国家全面进行统计方法制度改革，对经济监控、统计将更加严格、规范。因此，适当调低经济预期增速，是为了更切合我市发展实际，也是为了腾出更多的空间抓好转方式调结构，提高经济发展质量和效益。

为此，我们必须认识新常态，适应新常态，引领新常态。今年要重点做好以下工作：

（一）围绕提质量增效益，加快产业结构调整。

全力推动工业发展。坚持把工业发展摆在经济工作的首要位置，着力解决重点企业生产经营难、重大工业项目建设慢、企业培规难、工业园区基础设施薄弱等问题，重点实施好“四大会战”，促进工业转型升级。力争规上工业总产值达570亿元，增长10%左右。一是优化提升传统产业。加大技术改造投入，引导企业深化对信息技术和智能制造的应用，促进工业化和信息化融合发展。继续淘汰落后产能，淘汰水泥产能8.8万吨、铁合金产能2万吨。电力业方面，加快打造“热电联产”循环经济工业园，扩大用汽规模；用好铝电结合政策，提高发电负荷，增加上网电量。冶金业方面，研究出台帮扶政策，重点推进广铁公司企业改制，促进企业正常生产。制糖业方面，加快创建糖料研发中心，做大做强糖业综合利用产业。铝业方面，重点落实好铝电结合方案，确保铝产业上下游企业满产达产，着力引进铝精深加工项目。二是大力培育机械制造、茧丝绸、碳酸钙三大新兴产业。促进新兴产业向高端加工、高附加值方向发展。重点配套柳州汽车（机械）产业，狠抓象州捷和科技公司新能源电动车、康斯机械汽车零部件等36个已落地项目建设。大力推进象州华象丝绸染整、忻城缫丝真丝面料加工园等项目建设，打通茧丝绸及纺织服装加工核心环节。加快碳酸钙项目建设，着力打造碳酸钙产业基地。加快高新技术产业园扩园工作，抓好科技孵化中心建设，争创2家以上自治区级企业技术中心或研发中心。三是强力推进工业项目建设。重点解决工业用地等瓶颈问题，抓好合山发电公司“上大压小”第二台机组、华润水泥（武宣）公司二期、敏诚矿业公司二期技改等一批重点项目建设。四是提升园区承载能力。进一步完善园区供水、污水、路网等基础设施。完善园区规划，明确园区定位，每个园区培育1~2个重点产业。重点打造河南工业园和来华高新技术产业园。推进武宣县、象州县工业园区产城互动试点。五是提高服务企业水平。继续实施服务企业（项目）直通车活动，抓好中小企业信贷引导资金的融资工作，加大对重点工业企业帮扶力度，促使企业稳产达产，力争主营业务收入超亿元企业达108家。

加快推进现代农业发展。坚持“稳粮、保蔗、增蚕、强林果、兴养殖”的思路，着力解决农业产业结构单一、质量效益不高、服务体系不完善等问题，力争农林牧渔业增加值增长3%。一是大力调整农业产业结构。稳定现有粮食种植面积，保持甘蔗支柱产业地位，调减速生桉种植面积，大力发展桑蚕、水果、茶叶、蔬菜、水产畜牧等特色产业，力争新增桑园2万亩、特色水果8万亩、高标准茶园1万亩，推进泰国正大集团100万头生猪产业化、广西长岛集团2万头优质肉牛养殖屠宰加工等重大畜禽养殖和肉类加工项目建设。二是加快推进农业标准化、规模化、集约化。每个县（市、区）创建1个自治区级现代农业（核心）示范区。认真实施总投资6.12亿元的武宣、忻城、象州三县整县推进高标准基本农田整治项目。抓好11万亩糖料蔗“双高”基地建设。加强龙头企业、专业合作社、家庭农场规范化建设。完善农村土地流转机制，促进农业适度规模经营。三是健全农业服务体系。开工建设市农科所业务用房，建立农业科研示范基地。完善农业技术推广、农业综合执法、农产品质量安全监管、农产品流通等体系。加快创建全国“平安农机”示范市，提升农机服务水平。

大力推进现代服务业发展。着力解决商贸物流基础设施落后、消费市场活力不足等问题。一是促进商贸物流业发展。统筹推进城乡商贸物流网点建设，重点推进桂中建材市场、桂中家具城、桂中汽贸园等专业市场建设。支持第三方物流发展，培育来宾市煤炭交易中心等专业物流企业。启动现代服务业集聚区建设，重点培育来宾市宾港物流、来宾义乌小商品批发市场、华侨投资区高新技术产业园科技服务和金秀健康养生等4个集聚区。二是积极引导和促进社会消费。加快电子商务发展，积极培育消费热点。加强乡镇农贸市场建设，挖掘农村消费市场，释放城乡居民消费潜力。创新促进消费形式和内容，积极发展连锁经营、网络运营、物流快递等新型服务产业，大力培育休闲保健、法律咨询等新兴服务业。三是加快旅游业发展。抓好特色旅游名县、名镇、名村建设，推进“广西特色旅游名县”创建工作，力争金秀县成功创建、忻城县进入创建县行列、合山市进入备选县行列；抓好金秀大瑶山通用机场、莲花山旅游养生度假区、忻城薰衣草庄园、象州古象生态温泉二期等重点旅游项目建设。力争接待游客1500万人次以上，总收入突破100亿元。

（二）围绕增强发展后劲，加快推进项目建设。

发挥投资对促进经济增长的关键作用，力争完成固定资产投资470亿元。统筹推进247个亿元以上重大项目，完成年度投资150亿元。

基础设施项目方面。抓好水利项目建设。加快桂中治旱乐滩灌区一期工程建设，力争总干渠主体工程完工，完成年度投资6亿元，实现二期工程开工建设；抓好下六甲水库引水灌区补水工程前期工作；加强小型农田水利、水库除险加固等工程建设，完成投资4亿元。抓好铁路项目建设。加快推进湘桂铁路来宾段电气化改造、柳肇铁路来宾段及来合铁路城区段搬迁工程前期工作。抓好公路项目建设。大力推进柳州至武宣、来宾至马山、梧州至柳州、柳州至南宁高速公路改扩建及来宾绕城高速公路项目建设，实现来宾至马山、柳州至武宣高速公路主体建成；积极推进贺州至来宾、柳州经忻城合山至南宁等高速公路前期工作；加快推进寺山至贵港、象州（石龙）至来宾等6条二级公路建设；力争实现全市建制村全部通沥青（水泥）路。抓好水路项目建设。加强西江黄金水道基础设施建设和航道整治，推进兴宾港区莆田作业区、象州港区钓鱼公和猛山作业区、忻城北巷码头等6个港口项目建设，力争港口吞吐量突破1200万吨。

产业项目方面。重点推进来宾洁神新型锂动力电池、缘宏铝业公司汽车铝轮毂等90个续建项目建设；努力实现来宾绿翔公司天然植物提取及食品添加剂生产、桥宏公司特种纸和生活用纸生产等37个项目竣工投产；推进来宾天一纸业轻涂纸生产、东方昆仑新能源公司LED灯具生产等52个项目实现新开工；扎实做好鑫鼎铅锌锭冶炼及深加工、合山虎鹰循环经济综合利用、忻城石材产业集控区建设等68个项目前期工作，力争更多项目列入国家和自治区“十三五”规划项目盘子。

（三）围绕改善人居环境，加快城镇建设步伐。

完善城区功能。加大旧城改造力度，重点对柳来路、北四路、新兴北路、南一路、中南路、北二路、大桥路等9条道路，进行橡胶沥青路面铺设、人行道街砖铺装、雨污管网建设等方面改造，对北四路休闲广场、人民公园景观进行提升改造。继续推进老汽车站、老火车站等5个片区旧城改造。实现河西污水处理厂、来宾北站配套工程、图书馆、档案馆、群众艺术馆等项目竣工。全面完成桂中水城渠道建设，推动滨江公园、翠屏山公园、华侨大道和桂中大道延长线道路绿化景观提升改造。

启动新型城镇化综合试点工作。以我市列入国家新型城镇化综合试点城市和6个镇入选全国重点镇为契机，积极探索建立农业转移人口市民化成本分担机制，丰富城镇建设内涵，建设重点由规模扩张向质量提升转变。推动房地产业稳定健康发展。抓住自治区实施“百镇扶持壮大建设示范工程”机遇，引导凤凰、迁江、桐木、石龙、东乡、红渡等6个镇向特色小城镇转型。积极推进特色名镇名村建设，打造富有民族风情和地域特色的美丽村镇。启动城市总规修编、兴宾区行政区划调整工作。深入开展村镇规划集中行动，力争完成50户以上村屯的规划编制。进一步推进撤乡设镇工作。

提升城市管理水平。深入实施“城市经营管理年”活动，加大执法力度，严厉查处城乡结合部、背街小巷等重点区域的违法占地和建设行为。规范市容环境管理，大力整治“脏、乱、差”和粉尘污染，营造洁净舒适的人居环境。加快智慧城市建设，抓好数字化城市管理中心运行，完成“数字来宾”验收工作。加大城市道路交通秩序整治力度。

（四）围绕“双核驱动”战略，继续扩大开放合作。

全力融入珠江—西江经济带开放开发。按照“水路和陆路、码头建设和园区建设、工业发展和商贸物流发展、经济发展和生态保护”四个结合原则，加大对外开放合作，统筹区域资源优势，优化沿江产业布局，主动向粤港澳等先进生产力地区靠拢，承接产业转移。加快出台我市西江经济带发展规划，抓紧实施列入西江经济带基础设施建设大会战的河南工业园莆田路二期工程、凤凰工业园区路网工程等11个重大项目建设，推进我市列入 “双核驱动”战略的91个重大项目建设，特别是近期要实施的桥巩水电站至桂平航道整治工程、迁江工业园铁路支线等20个项目。

全力抓好招商引资。着力破解园区基础设施不完善、项目用地紧缺、审批时间过长等瓶颈问题，营造良好招商环境。创新方式，拓展招商渠道，加强与发达地区信息平台的合作，宣传推介我市招商环境，推进委托招商、以商招商和网络招商。围绕产业结构调整和转型升级，在依托现有资源、做强做优传统产业的基础上，突出抓好电子信息、新能源、汽车（机械）制造及零配件、先进加工制造业、现代农业及绿色食品、桑蚕及茧丝绸深加工（纺织）、生物及民族医药、新材料、现代服务业等九大重点产业招商，实现招商引资到位资金345亿元以上。

全力提高对外交流合作水平。借助中国—东盟博览会、商务与投资峰会等平台，进一步加强与东盟国家的交流与合作。加快推动柳来河一体化项目建设，搭建产业合作平台，积极承接柳州产业转移，主动融入南柳经济走廊。充分利用厅市战略合作、中国无机盐工业协会等平台，加强交流与合作，提升产业发展水平。

（五）围绕美丽来宾建设，狠抓绿色生态发展。

加强植树造林和生态保护。巩固“广西森林城市”创建成果，加快推进“国家森林城市”创建步伐。加快郊野公园、森林公园、湿地公园建设，重点打造城市及周边主要通道绿色林带，力争城市绿化覆盖率达40%。完成植树造林15万亩，实施337个绿化示范村建设。支持金秀县水源林和大瑶山自然保护区生态保护、忻城县石漠化治理、合山市矿山生态修复工程。力争森林覆盖率达51.3%。

强化节能减排。完善污水处理设施，确保迁江、凤凰污水处理厂等重点工程投入运行。加快推进规模化畜禽养殖减排项目建设。健全工业节能降耗预测预警和应急调控机制，严厉查处蓄意污染环境行为。引导企业申报节能技改、循环经济和清洁生产项目，推动工业企业向节能型、节水型、环保型方向发展。全面完成“十二五”和自治区下达的节能减排任务。

推进“美丽来宾·生态乡村”建设。全面开展“村屯绿化”“饮水净化”“道路硬化”三个专项活动，改善农村生产生活环境。积极开展自治区级生态村创建工作。

（六）围绕提高幸福指数，加快社会事业发展。

继续优先发展教育。深入实施教育“十大工程”。启动城区学校新一轮“四个二”（两所幼儿园、两所小学、两所初中、两所高中）工程建设，有效解决城区“大班额”“入学难”问题。实施新一轮学前教育三年行动计划。推进各县（区）义务教育均衡发展，重点支持忻城县迎接国家级评估认定，武宣县迎接自治区评估验收。推进普通高中多样化、特色化发展，提升办学水平。加快发展现代职业教育，重点支持市职教中心学校转型升级，确保创建国家中等职业教育改革发展示范学校通过验收，推进县级中等专业学校综合改革。扩大高等教育资源，加强内涵建设，促进转型发展。

推进科技文化体育事业发展。深入实施创新驱动战略。大力实施发明专利倍增计划，促进科技企业孵化器项目升级运行，加大自治区级农业科技园区建设力度，推进来华投资区申报自治区级高新技术产业开发区。抓好文化产业和文化事业发展。巩固提升我市作为全国首批“国家公共文化服务体系示范区”创建成果，完善公共文化服务体系，深入推进国家基层综合性文化服务中心建设试点工作，创造更多经验服务百姓，完成市博物馆陈列布展并向公众免费开放，继续实施农村电影公益性放映和广播电视村村通工程，推进忻城县莫土司衙署申报世界文化遗产工作，抓好“扫黄打非”工作。巩固提升我市作为全国首批 “全国全民健身示范城市”创建成果，继续提升、探索体育惠民、体育产业发展路径、经验。完成全民健身活动中心建设并投入使用，启动市体育公园建设，深入实施“农民体育健身工程”和 “雪炭工程”，抓好群众体育和竞技体育工作。

加快卫生计生事业发展。有效推进卫生和计生合并后的资源整合，强化卫生计生工作。深入推进自治区卫生城市创建工作，切实抓好市妇幼保健院、市全科医生培养基地等一批市、县卫生基建项目建设，推进金秀县瑶医药发展。完善新农合信息系统建设，确保参合率达96%以上。优化基本公共卫生服务，强化对艾滋病等重点传染病和重大传染病疫情的防控。严格贯彻执行计划生育基本国策，稳妥实施“单独两孩”生育政策，扎实开展国家免费孕前优生健康检查工作，综合治理出生人口性别比偏高问题，促进人口均衡发展。

提升社会治理水平。健全法治宣传教育和公共法律服务体系，深入推进涉法涉诉信访改革，加大矛盾纠纷排查化解力度，完善应急机制，依法处置各类突发事件，全力维护社会和谐稳定。加强社会治安综合治理，推进网格化管理，完善立体化治安防控系统，加大违法犯罪打击力度，提高公众安全感和满意度。落实“党政同责、一岗双责”责任体系，抓好安全生产。

（七）围绕增加群众福祉，切实保障改善民生。

提高城乡居民收入。继续实施农民人均纯收入倍增计划，大力实施粮食增产、菜篮子、生猪基地建设等农民增收“十二大工程”，提高农民经营性收入；严格落实农资综合补贴、良种补贴、农机购置补贴等各项惠农支农政策，提高农民政策性收入。健全工资收入分配制度，提高城镇居民收入，优化机关事业单位工资结构，推进事业单位实施绩效工资，合理调节企业工资收入分配，适时调整最低工资标准。全市居民消费价格指数涨幅控制在3.5%以内。

强化社会保障。完善和实施更加积极的就业创业政策，提升就业创业质量，实现城镇新增就业1.5万人以上，城镇登记失业率控制在4.1%以内。扎实推进城乡社会保障体系建设，强化社会保险基金征缴和监管，严厉打击劳动保障违法行为，抓好农民工工资清欠工作，健全新型社会救助体系。加快推进安置房建设，推行购建结合、以购为主的模式实施棚户区改造。切实做好库区移民安置工作。

扎实推进精准扶贫。重点推进列入自治区 “十百千”产业化扶贫示范项目建设，加强产业扶贫，完成甘蔗、茶叶、水果等经济作物新种和改造1.3万亩以上，减少贫困人口4万人以上。

办好民生实事。落实好自治区为民办实事工程，实施来宾市城区小街小巷硬化、城区小街小巷亮化、人民公园景观提升改造、城区环保公厕、村屯巷道硬化、百场演出进基层、城区中小学课程改革质量提升、全民健身活动中心二期、天网惠民、棚户区改造等10项为民办实事工程。

（八）围绕激发内生动力，全面推进改革步伐。

巩固提升重点领域改革成果。继续深化行政审批制度改革。认真实施总体方案及7项配套制度，建立健全监管措施和制度，加强事中事后监管；继续规范行政审批管理，完善行政审批事项目录管理，切实做好“接、放、管”工作；启动政府权力清单制度建设。深入推进城市综合管理体制机制改革，实质性运行城市管理指挥中心及城市管理执行系统、巡查系统、督查和评价系统。继续深化国有企业、工业区管理体制、华侨农场改革。全面推进农村土地承包经营权确权登记颁证工作。

抓好财政管理改革。贯彻落实新的《预算法》，深化预算管理制度改革。依法组织财税收入，加强重点税源管控和税收稽查。抓好财政支出，强化预算约束，推行预算绩效管理，加快预算支出进度。抓好节支管理，严格执行财政预算追加管理办法，规范和减少预算追加。加强结余结转资金管理，盘活财政存量资金，严格控制一般性支出，优先支持重点园区、产业、项目和重大民生项目建设，充分发挥资金使用效益。清理甄别政府性债务，规范债务管理，加大筹融资力度，化解债务风险。推广政府购买服务，降低行政成本。

推动金融服务改革创新。完善金融服务体系，引导和推动服务网点向县域、乡镇、村一级延伸。深化农村金融改革，强化金融服务“三农”发展。引导开发多元化金融产品和金融服务方式，拓宽抵押担保品范围，支持实体经济发展。推进小额贷款和融资性担保公司发展，力争新增小额贷款公司4家，融资性担保余额达12亿元。加快企业上市进程，力争完成2~3家中小企业股份制改造，1~2家上市培育企业在 “新三板”挂牌。

（九）围绕建设法治政府，全面推进依法行政。

提升依法行政水平。完善依法民主决策机制，健全行政决策风险评估、合法性审查、责任追究倒查等机制，提升依法科学民主决策水平。严格规范公正文明执法，坚持法定职责必须为、法无授权不可为原则，着力解决执法不规范、不公正、乱作为等突出问题。完善行政执法责任制，加强对政府部门特别是领导干部履职行为的监督，做到办事依法、遇事找法、解决问题用法、化解矛盾靠法，不断提升政府的执行力和治理能力。

依法推进政府职能转变。依靠法治凝聚共识，聚集力量，消除政府职能转变过程中的各种障碍，巩固各项改革成果。依法推进服务型政府建设，促进政府职能由行政指挥向综合服务转变，把政府经济管理职能转为宏观调控、市场监管和创造良好环境，正确运用政策引导、民主协商、信息咨询、经济发展规划、公共服务等手段，为经济社会发展服务。

推进政府廉政建设。强化行政权力制约监督，自觉接受市人大及其常委会依法监督和市政协民主监督，认真听取各民主党派、无党派人士、工商联、人民团体和社会各界人士的意见，主动接受人民群众和媒体监督，充分发挥监察、审计等部门的专门监督作用。严格遵守中央“八项规定”和国务院“约法三章”、党政机关厉行节约反对浪费条例，继续深化正风肃纪专项整治，扎实推进公车改革，加强“三公”经费管理，持之以恒纠正“四风”。坚持有腐必反、有贪必肃，严肃查处发生在工程建设、公共资源交易、行政审批、司法执法机关、国有企业等重点领域和关键岗位的腐败案件，认真查处发生在群众身边的基层干部“苍蝇式”腐败问题，以反腐倡廉实际成效取信于民。实施反腐倡廉“源头治理工程”，加强政风防控机制建设，健全政务公开制度，确保权力规范透明运行，树立政府清正廉洁的良好形象。

继续加强国防动员和后备力量建设，推动军民融合深度发展，广泛开展双拥共建活动，努力争创“全国双拥模范城”。依法保障公民特别是妇女、未成年人和残疾人的合法权益。充分发挥工会、共青团、妇联、文联、红十字会等群团组织的作用。继续做好外事侨务、民族宗教、统计、人防、气象、地震、水文、测绘、史志、档案、决策咨询、金融保险、邮政、电信等工作。同时，进一步理清新常态大背景下我市经济社会发展思路，谋划好“十三五”经济社会发展蓝图。

各位代表！推动科学发展、实现增长目标、建设生态来宾，任务艰巨，责任重大。让我们紧密团结在以习近平同志为总书记的党中央周围，在市委的坚强领导下，依靠全市各族人民，开拓创新、攻坚克难，为全面完成今年和“十二五”各项目标任务，打造广西“双核驱动”战略重要节点城市而努力奋斗！
